# Positive relationship between substrate-induced respiration rate and translationally active bacterial counts in soil

**DOI:** 10.1128/msystems.01009-25

**Published:** 2026-01-16

**Authors:** Nina Rose Camillone, Mary Ann Victoria Bruns, Raúl Román, Daniel Wasner, Estelle Couradeau

**Affiliations:** 1 Department of Ecosystem Science and Management, The Pennsylvania State University 8082, University Park, Pennsylvania, USA; 2 One Health Microbiome Center, Huck Institutes of the Life Sciences, The Pennsylvania State University 8082, University Park, Pennsylvania, USA; 3 Instituto Multidisciplinar para el Estudio del Medio Ramon Margalef, Universidad de Alicante 16718, Alicante, Spain; 4 Department of Agronomy, University of Almería 684448, Almería, Spain; 5 Department of Environmental Systems Science, ETH Zurich 27219, Zurich, Switzerland; East Carolina University, Greenville, North Carolina, USA

**Keywords:** soil microbes, microbial dormancy, microbial activation, soil respiration, BONCAT, flow cytometry, carbon cycling, substrate-induced respiration, soil health, cell enumeration

## Abstract

Soil microorganisms perform biogeochemical processes fundamental to soil functions. Bulk respiration, or microbial metabolic emission of CO_2_, is the classic indicator of soil biological activity. However, among the millions of microbes per gram of soil, only 0.1%–2.0% are metabolically active at any given time. Understanding the relationship between bulk soil respiration and microbial activity is complicated by microbes potentially awakening from quiescent states during incubation test periods. Here, we investigated this relationship through parallel measurements of substrate-induced respiration and translationally active cell counts using bioorthogonal non-canonical amino acid tagging (BONCAT). After a 6-h incubation of agricultural soil with glucose, galactose, or only water, active cell counts were positively correlated with respiration rates. As hypothesized, active cell numbers increased rapidly compared to total cell numbers after a 6-h incubation with glucose, suggesting newly activated cells. Additionally, carbon-amended soils respired more than water-only soils with similar active cell counts. This suggested that cells in carbon-rich environments were turning over freshly added carbon faster or metabolizing it less efficiently than those exposed to native substrates only. This study distinguishes for the first time microbial activation in the soil matrix using a translation signal, providing evidence that respiration rates reflect active cell numbers and varied metabolic responses, decoupled from cell growth upon soil wetting and carbon addition. We propose BONCAT as a useful tool to gain mechanistic insights into microbial activation and recommend combining it with tracking substrate incorporation and phylogenetics.

**IMPORTANCE:**

Many critical ecosystem services provided by soils rely on active microbes, even though most soil microbes are known to be quiescent or dormant much of the time. This study demonstrates that microbes become translationally active within hours after substrate addition and that the correlation between active cell numbers and soil respiration rates varies with the type of substrate. Advancing knowledge in this area will enable better interpretation of bulk soil respiration tests by land managers and inform modeling efforts that relate soil microbial respiration to global carbon dynamics.

## INTRODUCTION

Soil ecosystem services are intrinsically dependent on microbial activity through the many vital processes carried out by soil microbes. These include organic matter decomposition, plant nutrient provisioning, soil aggregate formation, nitrogen fixation, antibiotic production, and more (1). Eighty percent of the 3,200 Pg carbon in terrestrial ecosystems is stored in soil, with a flux of around 60 Pg per year due to mineralized CO_2_ loss via root and microbial respiration (2). Bulk soil respiration, or microbial emission of CO_2_ during metabolism, is a classic indicator of soil microbial activity and is commonly used to assess impacts of land use management on soil health and carbon cycling (3). Gaining insight into the roles of soil microbes in carbon cycling and ecosystem delivery, however, has been hampered by uncertainties in the relationship between soil respiration and the numbers of active microbes in soil (4).

Among the millions of microbial cells present in a gram of soil, most are quiescent, inactive, or dormant at any point in time (5), with 0.1%–2.0% metabolically active as a widely cited figure (6). Since soil microenvironments are typically low in carbon and energy sources (7), many microbes lower their metabolic activity to survive and then reactivate once resources become available (8). Higher soil microbial activity is considered to be associated with faster nutrient turnover, which supports productive aboveground/belowground ecosystems. However, microbial metabolic states between total dormancy and high activity are represented by a spectrum, resulting in multiple definitions of active microbes, diverging estimates of their numbers, and potential changes within relatively short time spans. Early studies enumerating redox-active soil microbial cells reported active fractions of 5%–20% or as low as 1% (4, 6, 9, 10). Other methods have found as many as 40% (11) and 50% (12, 13) active microbes in soil samples. More recently, an estimate of 90% active microbes in soil aggregates was based on the proportion of 16S rRNA amplicons that were labeled by H_2_^18^O-based DNA stable isotope probing (14). Clearly, active cell estimates in soils have depended on methods used. In the present study, we define active microbes as the cells that are extractable from soil and observed to undergo translation using bioorthogonal non-canonical amino acid tagging (BONCAT) (13, 15, 16). BONCAT involves short cell-tagging times (less than 2 h) and permits cell sorting of tagged and non-tagged cells.

The more process-oriented, bulk-soil approach for estimating soil microbial activity has been to measure soil respiration during incubation of unamended, pre-conditioned soil at favorable temperature and moisture conditions (3, 17). Respiration may also be measured after addition of a single, easily degradable substrate like glucose during substrate-induced respiration (SIR). The potential of a soil community to utilize a variety of substrates can be assayed using multiple substrate-induced respiration (MSIR), such as MicroResp (18). In this assay, CO_2_ evolution is estimated in an array of wells where soil is separately incubated with different carbon substrates. In any type of bulk respiration test, it is unknown whether evolved CO_2_ during the incubation period comes from already-active cells, cells undergoing reactivation, dying cells, or dividing cells. Since it is well established that substrates stimulate soil respiration to a greater extent than cell division (19), MicroResp assays utilize a standard 6-h incubation period, based on the assumption that cell replication will be negligible prior to measuring substrate use (20). This assumption was checked in one study, where high-throughput sequencing of most soil-substrate combinations found no change in 16S rRNA gene copy number within 6 h (21). Nevertheless, in any bulk soil respiration test, it remains unknown whether reactivation of dormant microbes contributes significantly to respiration or if respiration is controlled by a small percentage of already-active cells and their replication potential.

This uncertainty is a major limitation for whole microbial community analysis in understanding and predicting the main microbial actors in soil nutrient cycling. One approach to answering this question is to apply parallel measurements of bulk community activity and numbers of active microbes over time. This approach allows tracking of the relationship between overall respiration and numbers of active and reactivating microbes.

To investigate how an SIR response may be linked to increases in active cell numbers, we enumerated active cells using BONCAT. We posited that the relationship between bulk respiration and numbers of active cells is affected by the composition of available C substrates. Thus, as in MSIR, we used two carbon substrate treatments with different utilization pathways (glucose and galactose) to induce respiration, along with a water-only treatment. We measured bulk respiration and enumerated BONCAT-active cells after four incubation durations up to 24 h. We hypothesized that increases in average respiration rate among substrate treatments with increasingly longer duration times would reflect increases in the number of translationally active cells more than changes in total cell numbers. Increases in the numbers of translating cells over time would provide evidence that previously quiescent cells had become active. Additionally, we hypothesized that substrate treatments that differed in respiration rate would similarly differ in numbers of translationally active cells. These findings would suggest the importance of considering microbial activity status as a dynamic factor in modeling and managing soil ecosystem processes. Furthermore, if bursts in respiration are strongly linked with dormant cell activation, respiration tests can be developed as proxies for the potentially active microbial fraction, facilitating future collection and interpretation of soil microbial data.

## MATERIALS AND METHODS

### Soil collection and experimental design

All soil samples were collected at the Pennsylvania State University’s Russell E. Larson Agricultural Research Center near Pennsylvania Furnace, PA (40.72°N, −77.92°W), from a long-term tillage experiment established in 1978 (22). In 2018–2019, the mean soil organic carbon (SOC) for all no-till samples (*n* = 16) was 1.88 g kg^−1^, compared to 1.66 and 1.32 g SOC kg^−1^ for chisel-disked and moldboard-plowed samples, respectively (23). For the present study, a composite sample of bulk soil was collected in May 2023 (0–10 cm) from one no-till plot planted with wheat. The soil, classified as Hagerstown silt loam (fine, mixed, semiactive, mesic Typic Hapludalfs in USDA soil taxonomy), was sieved to 2 mm and stored at 4°C for less than 4 months before experimentation. Gravimetric water content was determined to be 16.6% by oven-drying a subsample at 105°C for 24 h. Available water holding capacity was determined to be 62.2% by the capillary method (24): a subsample was air-dried at room temperature for 3 days, placed in a pre-weighed setup of a wetted filter paper in a funnel, and allowed to wick up water for 1 h or until the entire surface of the soil glistened. Incubations of soil subsamples with cycloheximide to inhibit fungi or chloramphenicol to inhibit bacteria for 6 h showed that respiration was around 60% attributable to bacteria and 40% to fungi (Table S1) (25).

**Fig 1:**
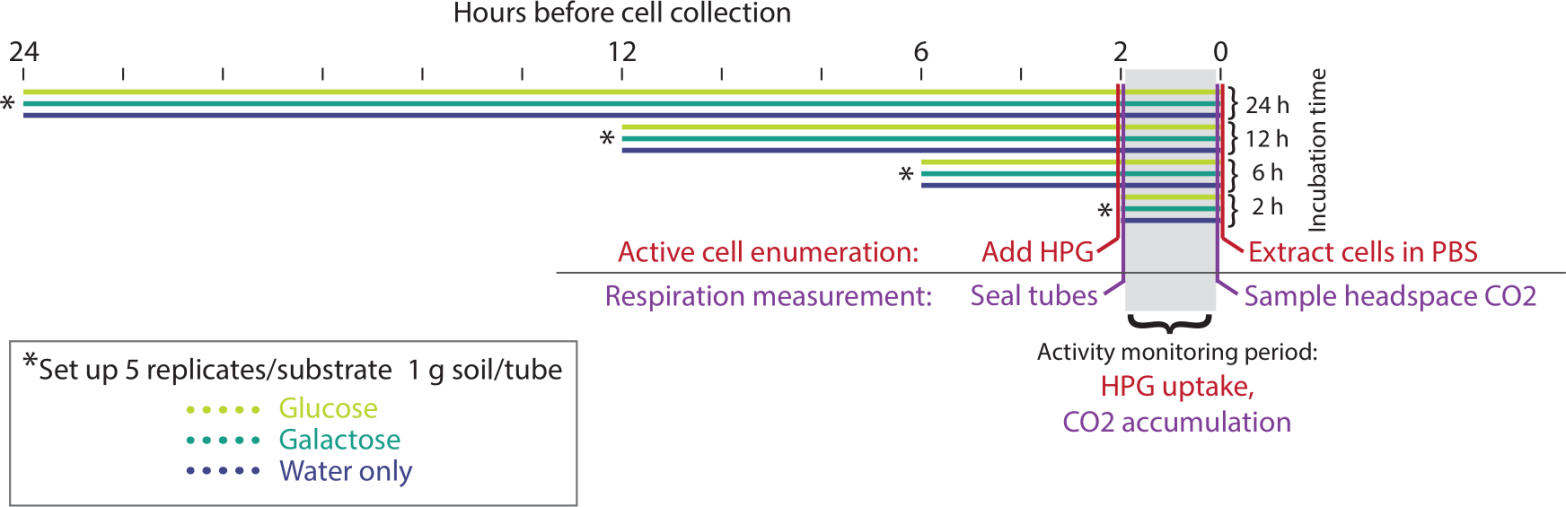
Experimental design as a timeline of substrate incubations, headspace sampling, and cell collection. L-homopropargylglycine (HPG) is a synthetic amino acid homologous to methionine used to label translationally active cells in the BONCAT workflow.

The experimental timeline is summarized in Fig. 1. Each experimental replicate consisted of 1.2 g field-moist soil (1 g oven-dried soil equivalent). Replicates were placed into 12 mL glass tubes sealable via screw-on caps with chlorobutyl rubber septa (Exetainer12ml Vial-Flat Bottom 738W; Labco Limited, High Wycombe, UK). The septa also allowed needle syringe access for headspace sampling. For substrate treatments, 100 µL of liquid was added to each tube: either an aqueous substrate solution containing 3 mol L^−1^ carbon (in the form of glucose or galactose; Sigma-Aldrich, Saint Louis, MO, USA) or ultrapure water. This resulted in a final concentration equivalent to 30 mg glucose g^−1^ soil water, which is the concentration commonly used with the MicroResp system (18, 20). The gravimetric water content of the soil after addition of the substrate was 30%, which was 50% of the available water holding capacity. The tubes were sealed to limit moisture evaporation and incubated in the dark at 22°C for 2, 6, 12, or 24 h. The time intervals reflect previous studies where respiration rates in surface horizon soil microcosms amended with glucose peaked from 5 to 10 h (depending on glucose concentration) and returned to near initial rates after 24 h (26). Galactose was selected as a substrate treatment because it stimulates a soil respiration rate intermediate to those of glucose and water-only treatments (18). Initial pilot experiments included lignin and vanillic acid as additional substrates, but these treatments resulted in very low BONCAT activity levels (on average less than 0.5%) compared to the false-positive discovery rate (0.2%) even after 4 h incubation. These substrates were not included, and further research will be necessary to understand why their BONCAT signal was so low. Possible explanations include metabolism by filamentous microbes not extracted by our methodology or chemical interference with BONCAT labeling. Six replicate tubes were included per substrate treatment per incubation length.

### Activity monitoring: respiration measurement and cell extraction

Two hours prior to the end of the incubation period (i.e., concurrently with the start of the substrate treatment for the 2-h replicates), the tubes were opened, flushed with ambient air, and resealed to begin a CO_2_ accumulation period of equal length for all replicates. While the tubes were open, 250 µL of 800 µmol L^−1^ L-homopropargylglycine (HPG), a synthetic amino acid homologous to methionine, was also added to five replicates per substrate treatment per incubation length. The sixth replicate in each group received 250 µL of ultrapure water as a control for the BONCAT dye signal. As a result, the moisture content of all replicates reached 90% of the available water holding capacity. The addition of HPG initiated the first stage of the BONCAT active cell enumeration method, where translationally active cells incorporate HPG into their proteins such that they can later be distinguished from HPG-negative inactive cells (13).

Two hours later at the conclusion of the incubation period, 2 mL of headspace air was collected with a needle syringe from each tube and immediately analyzed for CO_2_ content using a LI-7000 CO_2_/H_2_O Gas Analyzer (LI-COR). A calibration curve based on standards with known CO_2_ concentration was used to determine micromoles of CO_2_ in experimental samples. The CO_2_ content of the ambient air was recorded and later subtracted from the headspace concentrations to calculate the soil respiration rate. Bulk respiration was later calculated by dividing this corrected headspace CO_2_ content by the mass of soil in each replicate. Immediately after headspace sampling, 5 mL 0.02% Tween 20 in phosphate-buffered saline (PBS) was added to each replicate, and tubes were shaken horizontally for 5 min at high speed to extract cells from the soil. At this point, a killed soil replicate was added to the workflow. This subsample had been autoclaved three times at 1-day intervals (15 min, 121°C) in order to provide a control for staining with no cells. After shaking, the tubes were centrifuged for 5 min at 30 × *g* to separate soil particles without pelleting cells. The supernatant cell suspensions were stored at −20°C in 1 mL aliquots to which 500 µL 30% glycerol was added (final concentration 10% glycerol) and were processed within 1 week. Three replicates (one each from 6-h glucose, 12-h glucose, and 6-h water) had to be removed from analysis because respiration rates could not be quantified due to leaky tube seals.

### Active microbial cell labeling

The methods for collecting cells and BONCAT staining with a click chemistry reaction were adapted from previously published methods (13, 27). After thawing and briefly vortexing the frozen cell suspensions, they were centrifuged at 50 × *g* for 5 min to pellet any larger remaining soil particles. Previous studies have used much higher centrifuge speeds while maintaining high cell recovery (28), but for this soil, we had very low cell yields when higher speeds were used to pellet soil particles, perhaps due to cell clumping. The supernatant was recovered and passed through a 35 µm filter. Then, cells were pelleted from the suspension by centrifuging at 16,000 × *g* for 5 min. The supernatant was poured off and discarded, and the cells were resuspended in 70.4 µL 1× PBS in 2 mL snap-cap tubes by briefly vortexing. A dye mixture was prepared and added to each tube for a final reaction concentration of 5 µmol L^−1^ amino guanidine hydrochloride, 5 µmol L^−1^ sodium L-ascorbate, 100 µmol L^−1^ copper sulfate pentahydrate (Thermo Fisher Scientific, Eugene, OR, USA), 500 µmol L^−1^ tris-hydroxypropyltriazolylmethylamine, and 5 µmol L^−1^ FAM picolyl-azide dye in a final volume of 80 µL. Unless otherwise noted, all BONCAT reagents, including HPG, were purchased from Click Chemistry Tools (Scottsdale, AZ, USA). The tubes were incubated in the dark for 60 min to permit the click reaction between the azide dye and the HPG incorporated into the proteins of translationally active cells. Next, samples were washed to remove excess dye by centrifuging at 16,000 × *g* for 5 min, resuspending in 1 mL 1× PBS by brief vortexing, and repeating these steps twice more. The resulting cell suspension was diluted in 1× PBS and passed through a 35 µm filter (Falcon Round-Bottom Tubes with Cell Strainer Cap, 5 mL). SYTO 59 Red Fluorescent Nucleic Acid Stain (Thermo Fisher Scientific, Invitrogen, Eugene, OR, USA) was added as a counterstain to distinguish cells from clay particles. Cell suspensions were incubated in the dark for 15 min.

### Flow cytometry

Cell enumeration was performed using the LSR Fortessa flow cytometer (BD Biosciences, Franklin Lakes, NJ, USA) at the Pennsylvania State University Flow Cytometry Core, which is equipped with 405 nm (violet), 488 nm (blue), 532 nm (green), and 640 nm (red) lasers. The BONCAT dye, FAM-picolyl azide (490 nm excitation, 510 nm emission), was captured in a green channel (“FITC”) off the 488 nm blue laser. The DNA counterstain, SYTO59 (622 nm excitation, 645 nm emission), was captured in a red channel (“APC”) off the 640 nm red laser. Forward and side light scatter were also collected for all samples. All samples were run on the low flow rate setting (which we measured to be 22.6 µL/min for our samples). Data were collected for 120 s. Knowing the flow rate of the instrument enabled calculation of the number of cells extracted per mass dry soil using the number of cells detected and the volume of extract processed, recognizing that the extractable cells are a subset of the total soil microbial population (13).

As a negative control, a “cell extract” from a soil sample that had been autoclaved three times at 1-day intervals (killed control) was counterstained with SYTO59 to set the threshold for distinguishing cells from soil mineral particles. Similarly, soil samples incubated with water instead of HPG solution (HPG-negative control) were used to set the threshold for BONCAT-positive (BONCAT+) cells, since cells extracted from soil incubated with only water should not retain any of the BONCAT dye.

Data analysis was performed using FlowJo Software (Tree Star, Ashland, OR, USA). First, forward and side scatter fluorescence thresholds were set to constrain the size of particles examined. Second, a threshold for SYTO-positive (SYTO+) particles, which are assumed to be cells rather than clay particles, was determined based on fluorescence intensity in the APC channel from the killed (autoclaved) control samples counterstained with SYTO59. This step accounts for any potential autofluorescence from or staining of soil particles. Finally, a threshold for BONCAT+ cells was determined based on the fluorescence intensity in the FITC channel from the SYTO+ population (cells) in the HPG-negative control. To account for electronic noise from the flow cytometer or any potential retention of dyes on soil particles, the number of SYTO+ events detected in the killed control sample was subtracted from the SYTO+ count in other samples within each batch. Additionally, the number of BONCAT+ events detected in the HPG-negative control was subtracted from the BONCAT+ population in experimental samples. Any sample where the number of BONCAT+ events was less than or equal to the number of BONCAT+ events in the counterstain-only control was recorded to have zero active cells. Preliminary work using the LIVE/DEAD *Bac*Light Bacterial Viability and Counting Kit for flow cytometry (Thermo Fisher Scientific, Invitrogen) detected negligible percentages of dead microbes recovered using our extraction method (<0.65% on average; see Table S2), likely due to our cell extraction method failing to preserve cells without intact membranes, i.e., those deemed dead by the staining kit used.

Thus, we report total cells as representative of much of the living community.

### Calculations and statistical analysis

Statistical analysis was conducted using R version 4.2.1 (29), and figures were generated using R packages ggplot2 (30) and ggbreak (31). Total and active cell counts were compared across substrate treatments and incubation lengths using a generalized linear model with a negative binomial distribution due to overdispersion in the data (32). Pairwise comparisons of estimated marginal means were performed using the emmeans package in R (33). Incubation length was treated as a categorical variable due to the non-linear relationship of response variables and time. The rate of increase of active cell counts in each substrate treatment was calculated per unit of time as if it were following an exponential growth model for comparison with microbial growth rates reported in the literature. Our calculated rate of change of numbers of active cells represents the theoretical maximum growth rate if all new active cells resulted from cell division and no previously dormant cells became activated. Two-way ANOVAs with *post hoc* Tukey’s HSD tests were performed on whole-community and per-active-cell respiration data after Box-Cox transformations to improve normality using the MASS package in R (34). The relationship between respiration rate and log-transformed numbers of active cells was analyzed using linear regression. Equations for calculating total cells, active cells, and rate of change of active cells are included in the Supplemental material.

## RESULTS

### Cell counts and bulk respiration

**Fig 2:**
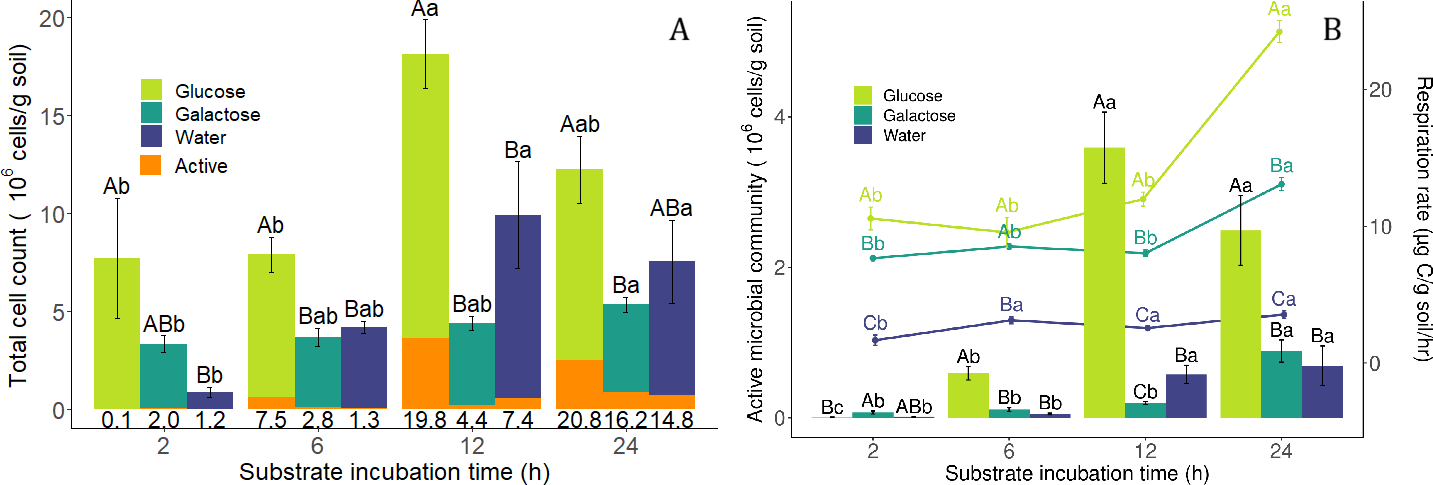
Total cells, active cells, and respiration rate. (**A**) Total cells over time for different substrate treatments, reported in cells per gram dry soil. The lower (orange) portion of each bar represents the active cell fraction. Numbers below each bar indicate the percentage of active cells. (**B**) Active cells (bars, left *y*-axis) and respiration rate (lines, right *y*-axis). Note that 0 for the right *y*-axis is elevated. For cell count data in panels **A** and **B**, letters represent statistically significant differences (*P* < 0.05) of estimated marginal means computed using a negative binomial regression model. For respiration data in panel **B**, letters represent statistically significant differences found by Tukey’s HSD test (*P* < 0.05) performed on Box-Cox transformed data. For panels **A** and **B**, uppercase letters reflect pairwise comparisons among different substrate treatments within the same time point, indicated by the *x*-axis, while lowercase letters represent statistically significant differences across time points within the same substrate treatment, indicated by color. Bars indicate standard error.

The total number of bacterial cells ranged from 3.1 × 10^5^ to 2.2 × 10^7^ g^−1^ soil throughout the experiment (Fig. 2A). The number of active cells ranged from 1.7 × 10^3^ to 4.4 × 10^6^ g^−1^ soil (bars in Fig. 2B, left *y*-axis), or 0.1% to 27.0% of the total (percentages noted below data bars in Fig. 2A). Generally, the number and percentage of active cells increased with time for all treatments. For the glucose treatment, numbers of total and active cells were highest after 12 h of incubation. In galactose, total and active cells were highest at 24 h. For the water treatment, total cells were highest at 12 h, while active cells were highest at 24 h. After 6 h, the increase compared to the 2-h incubations was statistically significant for active cells but not total cells in the glucose-amended treatment. For the other two treatments, neither the increase in total cells nor the increase in active cells was statistically significant at this point. Among the treatments, the glucose treatment had the highest total number of cells throughout the experiment, while the total cells in the galactose and water treatments never significantly differed from each other. As for active cells, they likewise were highest in glucose compared to the other treatments at 6, 12, and 24 h. In water, active cell numbers averaged slightly lower than in galactose at 6 and 24 h, but they were significantly higher than in galactose at 12 h. Contrastingly, galactose had the most active cells at 2 h, although this was still low at 2% of total cells. The rates of change for total cells were generally much lower than those for active cells (Fig. S1).

The bulk respiration rate increased after 24 h in all three treatments (lines in Fig. 2B, right *y*-axis). The respiration rate of the substrate-amended treatments did not differ significantly from their initial rates until 24 h. Glucose consistently had a higher respiration rate than galactose, and galactose consistently had a higher respiration rate than water-only treatment.

The largest absolute increases in numbers of total and active cells coincided with each other for all three treatments. In glucose and water, they were between 6 and 12 h, but in galactose, they were later, between 12 and 24 h. For glucose, the largest increase in respiration rate occurred between 12 and 24 h, after the large increase in number of active cells between 6 and 12 h. For galactose, the largest changes in number of active cells and respiration coincided between 12 and 24 h. For water, the largest increase in respiration occurred first, between 2 and 6 h, before the increase in active cells at 12 h. Glucose had the largest changes in total and active cells and in respiration rate compared to the other treatments.

### Relating respiration rate and active cell counts

Regression analysis revealed that respiration rates and active cell counts were positively correlated, although this relationship differed by substrate. Statistically significant relationships were found between these variables within each treatment and across all treatments together, with the marginal exception of the glucose treatment at a *P* value of <0.06 (see Fig. 4A; equations are reported in this figure). Respiration rates relative to active cell counts were highest in the glucose treatment, intermediate in the galactose treatment, and lowest in the water treatment. Generally, carbon-amended soils had higher respiration rates than water-only soils with similar active cell counts, mathematically evidenced by the water-only treatment’s lower regression coefficient (see Fig. 4A).

## DISCUSSION

### Methodological insights and comparisons

Our study documents for the first time activation of bacterial cell protein translation in the soil matrix following substrate amendment, presenting evidence of cell activation corresponding to elevated respiration without a proportional increase in total cell numbers, supporting our first hypothesis. We strategically applied the BONCAT method because it detects translational activity of existing ribosomes and requires 2 h or less as labeling time, providing a “snapshot” of currently active microbes within that specific time frame rather than a recap of which microbes were active throughout the entire incubation. We contend that a large portion of increased respiration during short-term incubations can arise from newly translating cells, although we do not exclude potential contributions to respiration made by replicating cells. Our study also shows that activation dynamics differ with the type of substrate added, supporting our second hypothesis. The greatest increases in active cells occurred between 2 and 6 h in soils treated with glucose and between 12 and 24 h in soils treated with galactose.

The preponderance of inactive or dormant microbes in soils has made it challenging to relate active microbial numbers to respiration, as inactive cells can reactivate during bulk soil incubations. In the present BONCAT study, active cells ranged from 0.1% to 27.0% of total extracted cells within a 24-h period (Fig. 2). In the literature, estimates made using diverse methods range from under 1% to over 90% (Table 1). This wide span may be due to natural differences between soils across time and space or inefficiencies in some methods compared to others. In contrast to these other methods, BONCAT offers the ability to label cells undergoing activation within the soil matrix but does require cell extraction for their detection. Drawbacks of other methods include the need for cell extraction prior to labeling or longer incubation times that do not permit capture of activation dynamics. Methods that use fluorophores like CTC, CFDA, and FISH (see abbreviations in Table 1) are applied on cell suspensions extracted from soil rather than microbes living in the soil matrix (5, 10–12). Direct counting in soil smears is typically impractical due to the small sample size and signal interference by soil colloids.

**TABLE 1:** Comparison of methods to enumerate active bacteria enumeration in soil, indicating the type of activity measured, time scale, labeling approach, and active cells as percentage of total cells^*a*^

Method	Activity type	Time scale	Labeling approach	Reported active fraction (%)
CTC	Aerobic respiration (redox)	30+ min	Extracted cells	2–6 (10) <1 (5)
CFDA	Metabolic enzymes	30+ min	Extracted cells	42 (11)
FISH	rRNA content	Fixed cells	Extracted cells	55 (12)
[^3^H]thymidine incorporation	DNA replication	48–95 h	Extracted cells	8–18 (35)
DNA-SIP	DNA replication	1+ days	In soil matrix	91 (14)
BONCAT	Protein translation	30+ min	In soil matrix	25 (13)

^*a*^ CFDA, 5(6)-carboxyfluorescein diacetate (a substrate hydrolyzed by intracellular esterases); CTC, 5-cyano-2,3-ditolyl tetrazolium chloride (an artificial electron acceptor); FISH, fluorescence *in situ* hybridization; SIP, stable-isotope probing.

Incorporation of [^3^H]thymidine is another common method for measuring soil microbial activity, but radioactivity of labeled cells must be measured in bulk, whether cells are labeled before or after extraction (35, 36). Cellular uptake of thymidine is rapid (<120 min), but not all soil bacteria are capable of incorporating it (37). Incorporation of stable isotopes like H_2_^18^O (heavy water) into microbial DNA or RNA is another means used to demonstrate growth of bacteria during incubations as short as 3 h but typically at least 24 h (38–41). After nucleic acid extraction from the soil, the heavier, labeled nucleic acid fraction can be isolated by isopycnic centrifugation for high-throughput amplicon sequencing. Changes in the proportions of specific sequences over time can provide information on which community members were actively growing; however, they would not capture cells that are translationally active but not duplicating their DNA in that time frame. Additionally, this set of techniques would not allow distinguishing which cells were already active at the onset of the incubation or reactivated during this 24-h period, while our data indicate that the community is shifting quickly in that time frame. By comparison, BONCAT can detect cell activation in shorter timescales because it detects translational activity of existing ribosomes (e.g., within 30 min of revival of *Bacillus subtilis* spores) (42). Due to its short labeling time, the BONCAT method can be coupled with frequent respiration measurements to discern the relationship between short-term CO_2_ release and newly translating cells.

Some remaining methodological challenges in active cell enumeration are specific to BONCAT, while others apply more broadly. For example, BONCAT does not detect the activity of microbes with access to native methionine (outcompeting analog HPG) or microbes with adequate protein reserves without need for translation. On the other hand, any method involving a probe raises questions regarding the impact the probe has on the community. In BONCAT, HPG is known to alter around 15% of metabolites in *Escherichia coli* (36). HPG addition could stimulate microbes by providing a needed amino acid, or it could impede functionality by inducing protein misfolding. While BONCAT labeling can be done over longer periods of time, we limited the HPG incubation to 2 h in this study to keep any positive or negative influence on the microbial community minimal.

Another consideration for studying soils, especially, is that extraction procedures do not recover all microbes present, particularly filamentous microbes such as fungi and *Streptomyces*, or cells strongly adhered to mineral surfaces. Typically, soils contain 10^7^–10^10^ prokaryotic cells g^−1^ (43), compared to counts in this study of 5 × 10^6^ g^−1^ after 2 h of incubation (Fig. 2). The latter counts were only 1% of numbers observed in suspensions of agricultural sandy loam soils shaken in water in the experiments of Bååth (44). These cell extracts had very low numbers of dead microbes (Table S2), which may contribute to an underestimation of total cell counts as previous research has reported 40% dead microbial cells in soil (6). Importantly, our data represent replicates of a single homogenized soil sample, keeping any biases consistent and allowing comparison of the extractable, unicellular microbial community across treatments, although any filamentous or biofilm life strategies are not represented.

### Glucose amendment reactivated dormant cells

**Fig 3:**
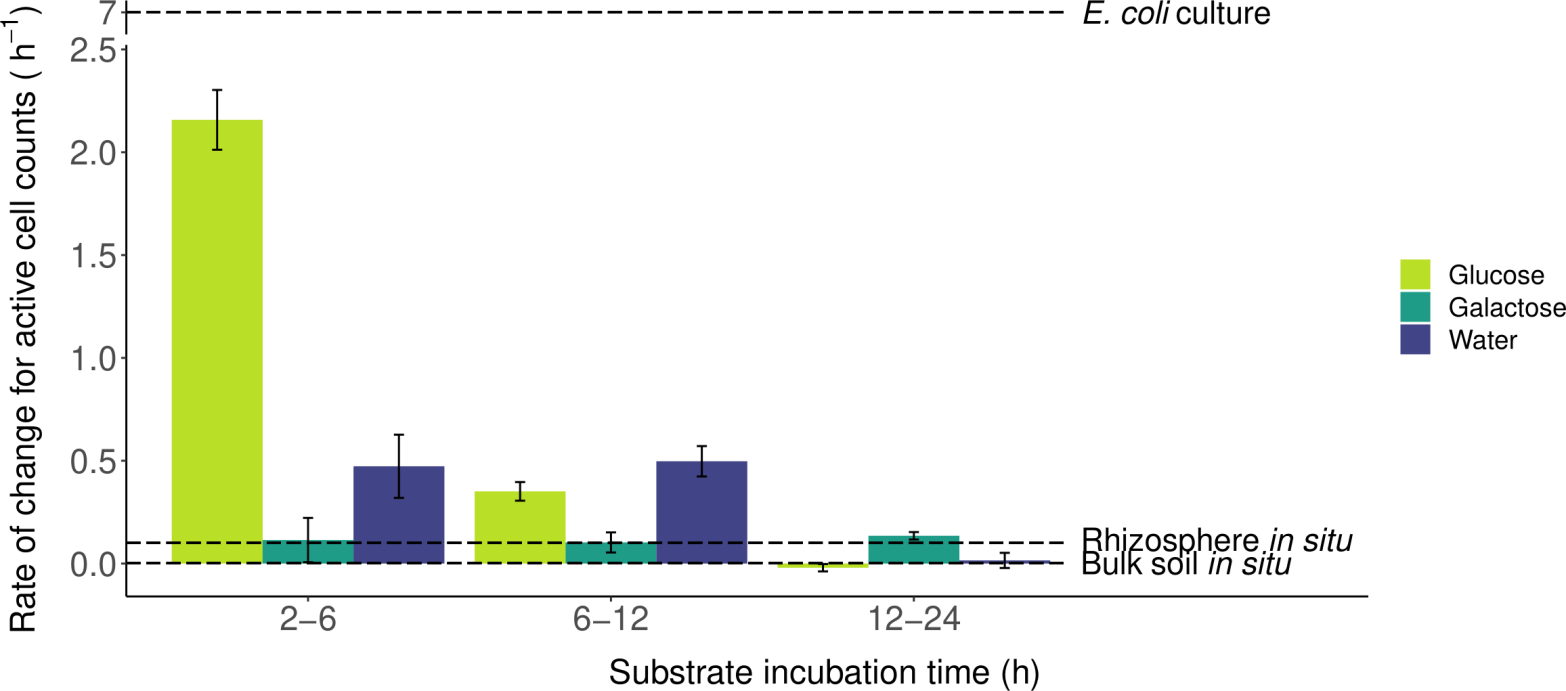
Rate of change in the number of active cells over time. Bars indicate standard error, propagated from the standard error in the enumerated community sizes. For comparison, dashed lines indicate several known growth rates: a laboratory culture of *Escherichia coli* (generation time: 20 min) (46), an active rhizosphere community (generation time: 7.2 h) (47), and bulk forest soil (generation time: 14.1 days) (49). Note the break in the *y*-axis between 2.5 and 7.0.

Comparisons among our calculated rates of increase of active cells and literature values of soil microbial replication in the soil matrix suggest that reactivation of dormant cells most likely contributed to our observed increase in active cells during particular time frames for some substrate treatments (Fig. 3). Specifically, the most rapid increase in active cell numbers was from 2 to 6 h of incubation with glucose. In comparing these two time points for glucose, the increase in active cell numbers surpassed the increase in total cell numbers. Without reactivation of dormant cells, already-active cells would have needed to replicate every 0.62 h (37 min) to achieve the observed increase through hypothetical exponential growth. This approaches the theoretical maximum replication rate under optimum soil conditions of a 0.5-h generation time (45). For comparison, *Escherichia coli* has a generation time around 0.3 h *in vitro* (depicted in Fig. 3 as rate of change per hour) (46). In the soil matrix, generation times for soil microbes have been measured to be as low as 7.2 h in the rhizosphere (47), 0.7–63.5 days for different taxa (48), and 14–45 days on average in bulk soil (49) (depicted in Fig. 3 as rate of change per hour). Similarly, more rapid rates of increase in active cell counts, compared to replication rates reported in the literature, suggest that dormant microbes were also reactivating in the water treatment from 2 to 12 h and the glucose treatment from 6 to 12 h (Fig. 3). The rate of change in active cell counts in the galactose treatment did not measurably differ throughout the experiment and remained comparable to a highly active rhizosphere environment. Since the increase in active cell counts can plausibly be attributed to cell replication, we do not report these numbers as evidence of dormant cell activation in the galactose treatment.

Previous literature reports that replication of soil microbial DNA can occur within 3-h laboratory incubations, indicating growth and potential reproduction (50). For this reason, we do not presume to attribute our observed increases in active cells to dormant cell activation alone. However, if cell replication of already-active cells in our glucose treatment at 2 h was comparable to that of an active rhizosphere (47), their exponential replication would account for less than 1% of the observed increase in active cell counts by 6 h. Thus, our calculations support a finding that the majority of additional active cells observed after 6 h of glucose incubation are attributable to dormant cell activation.

### The relationship between bulk respiration and active community size differed over time and by treatment

**Fig 4:**
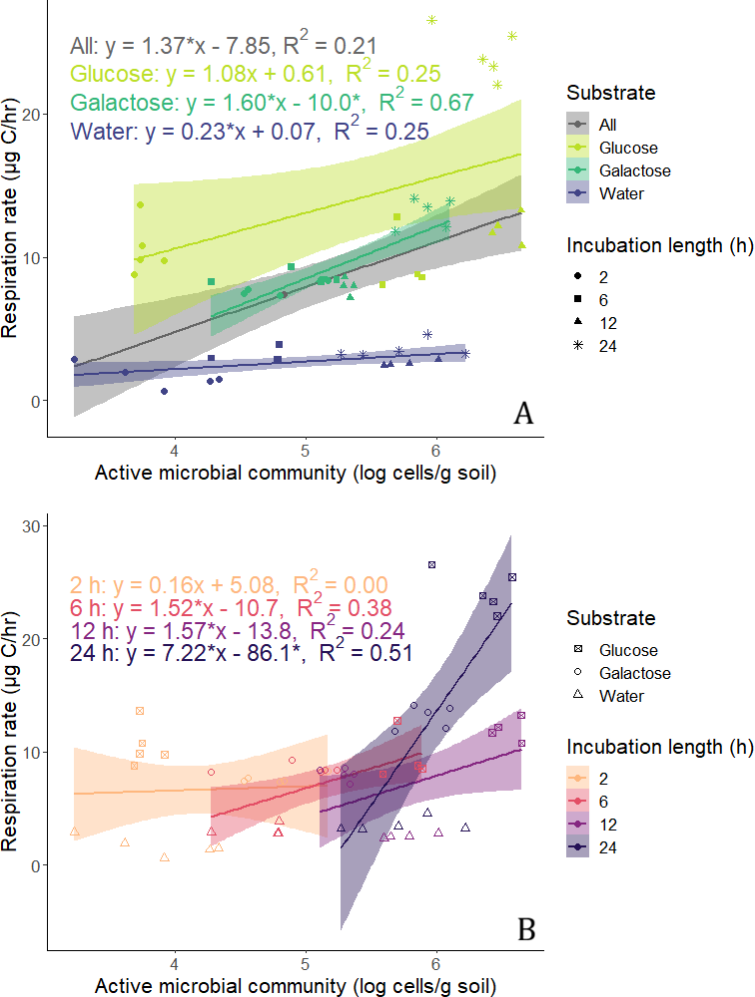
Regressions of bulk respiration onto active cell counts in log scale. The shaded regions show the 95% confidence region for each regression line. (**A**) Regression lines for all data and for each substrate individually. *P* values for coefficients and intercepts, respectively, are <0.001 and 0.08 for all data, 0.06 and 0.9 for glucose, <0.0001 and 0.009 for galactose, and 0.03 and 0.9 for water. (**B**) Regression lines for each time point individually. *P* values for coefficients and intercepts, respectively, are 0.9 and 0.6 for 2 h, 0.03 and 0.2 for 6 h, 0.08 and 0.2 for 12 h, and 0.003 and 0.008 for 24 h.

Based on the evidence for predominant cell activation at certain time periods provided in Fig. 3, respiration rates can be compared among already-active and newly activated microbial communities with similar active cell counts (i.e., similar *x*-axis coordinates in Fig. 4A). For instance, the water-only treatment at 24 h (no net cell activation) had lower respiration rates than the glucose treatment at 6 h (likely cell activation). This suggests that changes in translational activity status may contribute to the increased respiration rates observed for carbon-amended replicates.

Higher respiration and active cell numbers in the glucose treatment than the galactose treatment are consistent with the ability of cells to utilize glucose directly. By contrast, galactose requires multiple enzyme-catalyzed reactions before a downstream metabolite enters the glycolytic pathway (51). Additionally, glucose addition has a priming effect on soil stimulating the degradation of native organic matter (52), while it is unknown whether galactose would have a similar or opposite effect. The water-only microbial communities are unique among the treatments in their exclusive dependence on native soil carbon substrates. Chemical and physical association of these heterogenous molecules with mineral particles in soil can lead to low carbon availability despite its abundance, resulting in microbial carbon limitation (53). The microbial efficiency-matrix stabilization framework theorizes that a lower proportion of metabolized carbon is respired when inputs have low ratios of carbon to other nutrients and a high capacity for stabilizing association with soil minerals (54), as in our water-only treatment. Accordingly, our results suggest that the relatively carbon-poor soil environment supported an expanding active and total microbial community with minimal changes in respiration rate (Fig. 2B and 4A). This could be explained by more efficient carbon usage, i.e., prioritizing carbon assimilation as biomass relative to mineralization, due to high complexity among native available carbon substrates (55). Conversely, high respiration in carbon-rich environments may be driven by a decrease in carbon use efficiency, which has previously been attributed to excess carbon availability (56), although more rapid carbon metabolism could also explain this observation. Future research using isotopic tracers to measure carbon use efficiency can explore more concretely how complexity of available carbon substrates affects soil respiration relative to microbial growth and activity status.

### Lag between respiration response and increase in active cells

Regression models calculated within each time point indicated a positive relationship between respiration and active cell counts with statistically significant regression coefficients at both 6 and 24 h, but not at 2 or 12 h (Fig. 4B). Thus, at these two latter time points, we found weak or no correlation between respiration and enumerated active cells. One way of viewing these results is to consider “respiration per active cell” by dividing bulk respiration by the number of active cells for each replicate (Fig. S2). This is not an accurate measurement of the respiration of individual cells, especially because bulk respiration includes respiration of filamentous microbes and any others not captured by BONCAT. Instead, it allows respiration to be related to the size of the translationally active community and to estimate respiration per active cell. Using this simplified metric, we found that the substrate treatment with the highest respiration per active cell was glucose after 2 h and galactose after 12 h. These differences suggest that glucose-amended soil microbes began rapidly metabolizing with a burst of respiration per active cell within 2 h before the activation of dormant cells by 6 h. Meanwhile, galactose induced increases in respiration and active cell counts at a slower but steadier rate. In fact, the galactose treatment exhibited the least variation in average respiration per cell and the strongest positive correlation (highest regression *R*^2^) between bulk respiration and active cell counts (Fig. 4A). A low level of respiration from BONCAT-negative cells also would have contributed to a strong correlation between respiration and active cells. This could be the case if few cells already contained the enzymes necessary for galactose metabolism.

Regardless, for all substrate treatments, average respiration per active cell tended to decrease over time (Fig. S2). Within 2 h of substrate addition, the number of active cells was similar among treatments (Fig. 2), even though the two carbon-amended treatments already had much higher bulk respiration rates than water-only treatment (Fig. 2). Thus, our results indicate an initial lag where changes in the respiration response are decoupled from changes in translational activity, perhaps due to newly activating cells relying on already existing protein reserves before resuming translation. In the field, this could translate to increased carbon emissions from more frequently disturbed soils.

### Implications for soil testing

While respiration tests are already a common soil health metric taken to reflect microbial activity, this study adds to our understanding of the complex underlying states of the microbial community that combine to produce observed respiration rates. One complication is the spectrum of activity between the seeming dichotomies of dead/alive and active/dormant. Notably, potentially active microbes have been defined as those that resume activity after 4–12 h of stimulation (6). Soil testing already utilizes SIR for crop yield prediction with 6-h incubations (57, 58), consistent with this time frame. In the present study, results characteristic of potentially active microbes were observed in increased active cell counts but not total cell counts between 2 and 6 h of glucose incubation (Fig. 2). Thus, 6-h SIR tests may be interpretable indicators of potentially active, substrate-responsive microbes in agricultural soil. Using BONCAT’s short labeling times, future research can compare differently managed soils to determine whether the initially active fraction (e.g. 2-h incubation) or potentially active fraction (e.g. 4–6 h incubation) is more useful in explaining microbially mediated soil functions observed in the field. A soil with a presently low active fraction or field respiration rate could also have a strong potentially active fraction revealed by SIR that might better support diverse ecosystem services, compared to a soil with a consistently active, high-respiration microbial community rapidly depleting carbon stores, such as is characteristic under frequent tillage (59).

While our study does not aim to provide recommendations regarding the practical implementation of soil health testing, our findings provide useful context for interpreting respiration data in terms of the soil microbes and carbon substrates interacting to produce these test results. For the first time, our data demonstrate the substrate-dependent relationship between bulk soil respiration rates and the number of active microbes. Future studies can add detail by combining BONCAT and cell sorting and metagenomic sequencing, along with simultaneous measurement of growth rates using internal DNA standards (48) or the ^18^O-H_2_O method (49). An improved understanding of cell activation dynamics can enhance our ability to implement and interpret soil health tests used to inform soil management decisions. In light of our findings, we suggest that the goal of microbial soil management can be approached not only in terms of bulk microbial activity but also by considering the capacity of the potentially active microbial fraction to support desired soil functions.

## Data Availability

Original flow cytometry data are available as FCS files at doi.org/10.26208/G8KX-GG32 through Penn State’s DataCommons.

## SUPPLEMENTAL MATERIAL

Supplemental material msystems.01009-25-s0001.docx Supplemental equations, tables, and figures.

Supplemental plots msystems.01009-25-s0002.docx Flow cytometry plots.

ASM does not own the copyrights to Supplemental Material that may be linked to, or accessed through, an article. The authors have granted ASM a non-exclusive, world-wide license to publish the Supplemental Material files. Please contact the corresponding author directly for reuse.
