# Supplementary material for: Positive relationship between substrate-induced respiration rate and translationally active bacterial counts in soil
Source: mSystems. 2026 Jan 16;11(2):e01009-25. doi: 10.1128/msystems.01009-25 (PMC12911417; doi:10.1128/msystems.01009-25)
Supplement: Supplemental material — Supplemental equations, tables, and figures. [file msystems.01009-25-s0001.docx]

**Supplemental equations.**

Total cell counts per gram soil were calculated as follows:

$$\frac{\frac{sample {SYTO}^{+}count-killed control {SYTO}^{+}count}{analyzed extract volume x dilution factor}}{soil sample mass (g)}$$

Active cell counts per gram soil were calculated as follows:

$$\frac{\frac{sample {BONCAT}^{+}count-{HPG}^{-}control {BONCAT}^{+}count}{analyzed extract volume x dilution factor}}{soil sample mass (g)}$$

Rate of change in active cell counts (*r)* was calculated as follows for each consecutive pair of incubation lengths:

$$x_{t}=x_{0}{(1+r)}^{t}$$

Where $x_{0}$ is the number of active cells after the shorter incubation, $x_{t}$ is the number of active cells after the longer incubation, and *t* is the time difference between the incubation lengths (in hours).

**Supplemental table 1.** Respiration response to selective inhibition. Compounds were added to 1 g subsamples of soil along with glucose for a 6-h incubation, following the same glucose treatment and respiration measurement protocols described in the methods section. Water was added without HPG for the final 2 h of the incubation, during which CO_2_ was collected. Inhibition is reported as the percentage decrease in respiration relative to the soil with glucose and no inhibitory compound. Increased inhibition was not observed with higher dosages of the inhibitors (data not shown). Proportions of respiration attributable to bacteria and fungi were calculated by the equations (A-B)/(A-D) and (A-C)/(A-D) respectively (Anderson & Domsch 1974), where A is the uninhibited respiration rate, B is the respiration rate with streptomycin and penicillin addition, C is the respiration rate with cycloheximide addition, and D is the respiration with both inhibitor solutions. A - [(A-B) + (A-C)] was 5% different from D, the suggested acceptable allowance for experimental errors. The lower percent inhibition seen for the combined treatment than an expected additive effect of the individual inhibition treatments suggests some occurrence of non-specific inhibition.

| **Inhibitor** | **Dosage (µg/g soil)** | **Respiration inhibition** |
| --- | --- | --- |
| Cycloheximide (antifungal) | 500 | 17 ± 1% |
| Streptomycin and penicillin (antibiotics) | Penicillin: 150  Streptomycin: 250 | 20 ± 2% |
| Both antifungal and antibiotics | Same as above | 32 ± 7% |

**Supplemental table 2.** Percentage of live cells for select samples.

| **Substrate** | **Incubation length (h)** | **Live cell fraction (mean)** |
| --- | --- | --- |
| Water | 2 | 98.9% |
| Water | 12 | 98.9% |
| Water | 24 | 99.2% |
| Glucose | 2 | 99.1% |
| Glucose | 6 | 98.9% |
| Glucose | 12 | 98.9% |
| Glucose | 24 | 99.1% |


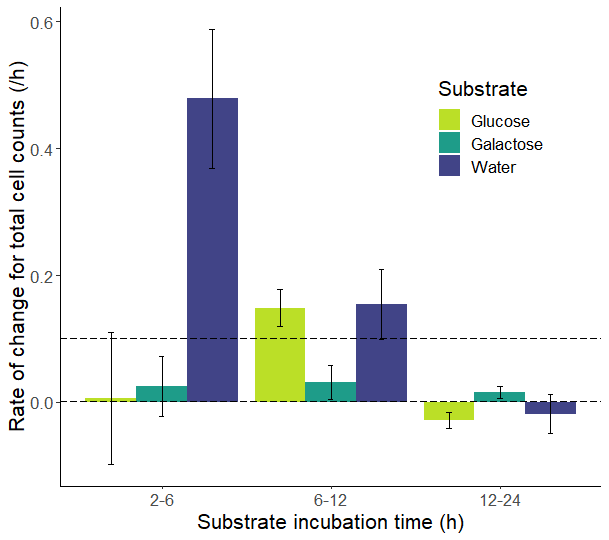


**Supplemental figure 1.** Rate of change of total cell counts, calculated as described for active cell counts in Figure 3 in the main text. The dashed horizontal lines indicate literature values for bacterial growth rates in bulk soil (lower line) and the rhizosphere (upper line).


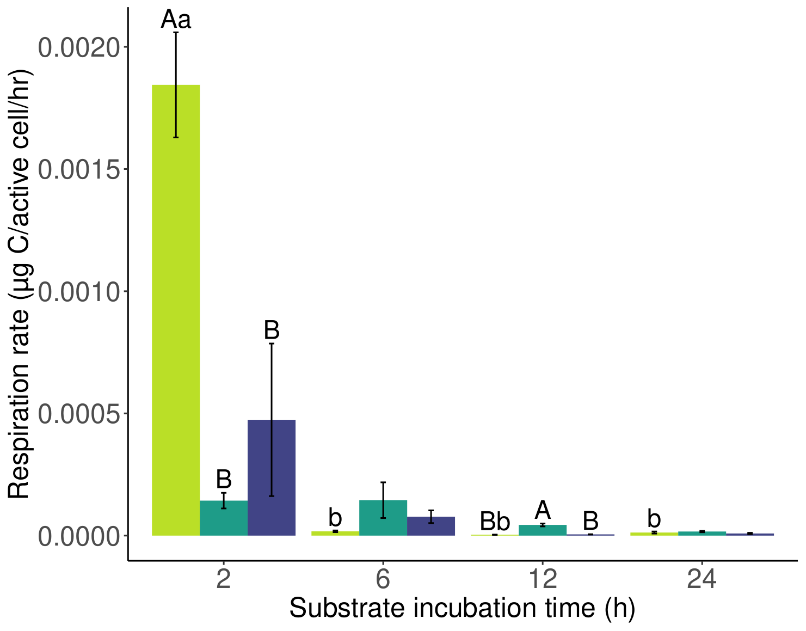


**Supplemental figure 2.** Observed respiration rate per active cell, calculated by dividing bulk respiration by the number of active cells. Uppercase letters represent statistically significant differences found by Tukey’s HSD test (p<0.05) among substrate treatments within the same time point, indicated by the x axis. Lowercase letters represent statistically significant differences across time points within the same substrate treatment, indicated by color. The absence of a letter of a particular case above a bar indicates that Tukey’s test was not conducted due to an insignificant ANOVA result. Statistics were performed on box-cox transformed data. Bars indicate standard error.
