# Supplementary material for: Positive relationship between substrate-induced respiration rate and translationally active bacterial counts in soil
Source: mSystems. 2026 Jan 16;11(2):e01009-25. doi: 10.1128/msystems.01009-25 (PMC12911417; doi:10.1128/msystems.01009-25)
Supplement: Supplemental plots — Flow cytometry plots. [file msystems.01009-25-s0002.docx]

**Flow cytometry plots.**

FCS-A on the x-axes refers to forward scatter. The y-axes labeled APC-A capture the fluorescence of the DNA dye SYTO 59 intended to separate cells from clay particles in the left-hand column plots. In the right-hand column, the y-axes labeled FITC-A capture the FAM-picolyl azide BONCAT dye present in active cells only. Numbers in the gate labels indicate the percentage of plotted events that are included in the drawn gate. In each pair of plots, the one on the right includes only events that were gated as SYTO+ in the plot on its left.

Each section starting with a “killed control” (autoclaved-sterilized soil) and an HPG-negative control represents samples from the same run on the flow cytometer that thus used identical thresholds to gate cells and active cells. The controls included at the beginning of each of these sections were used to correct for false positives. Sections that include the same control plots as each other were part of the same flow cytometry run.

**Water treatment**

Killed control


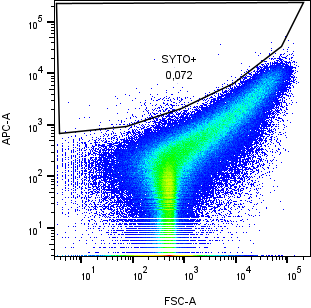

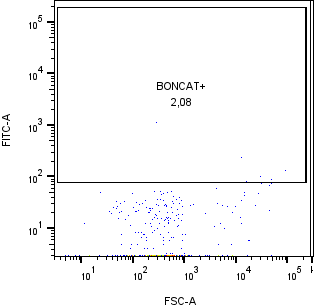


HPG-negative control (24-h water)


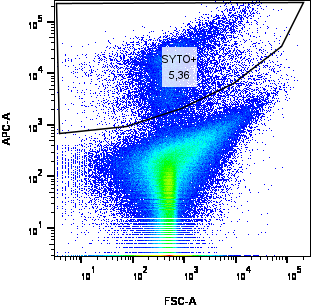

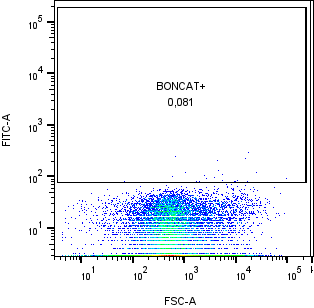


Experimental samples (2-h water)


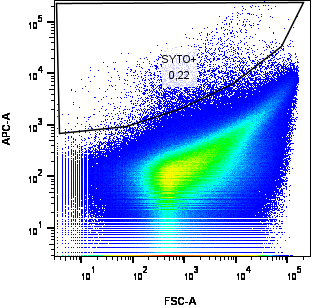

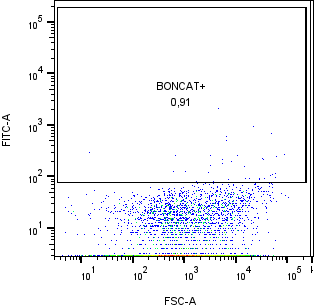


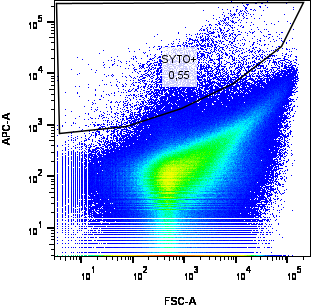

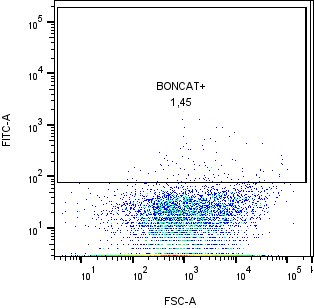


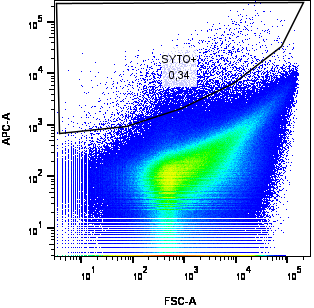

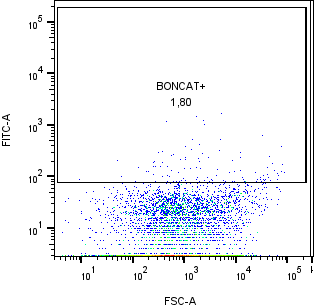


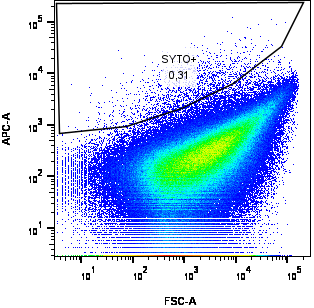

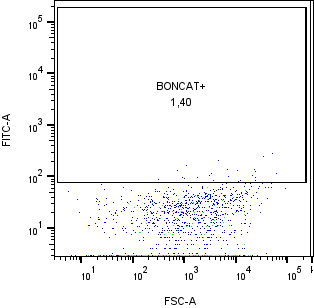


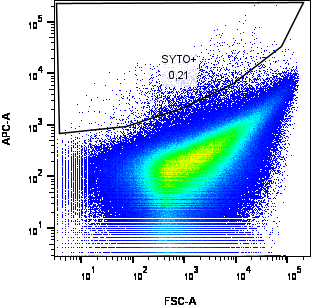

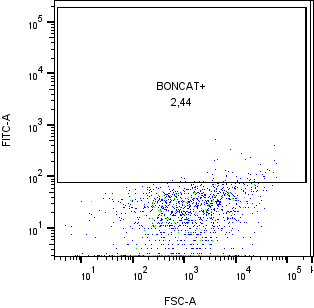


Killed control


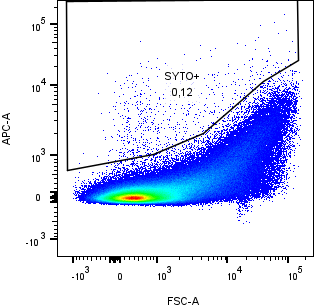

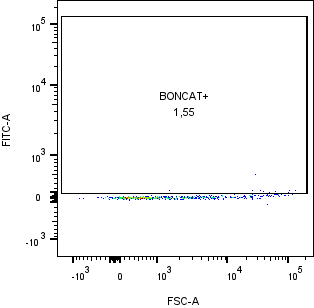


Syto control (24-h galactose)


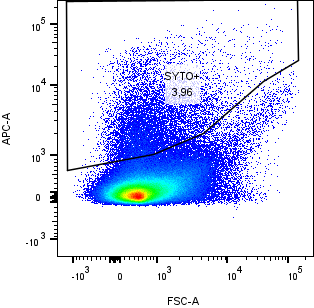

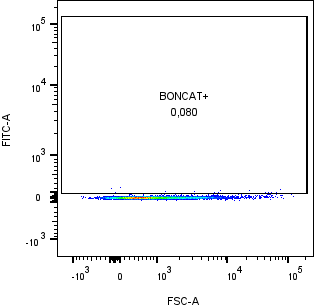


Experimental samples (6-h water)


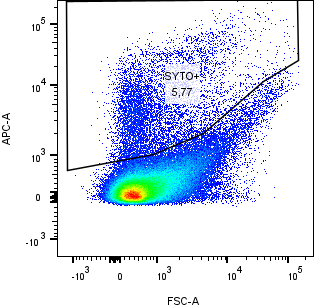

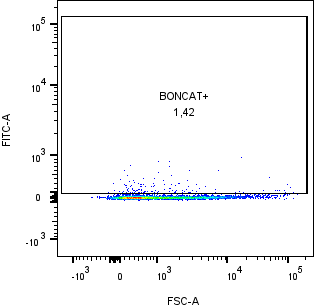


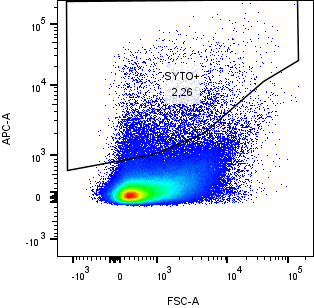

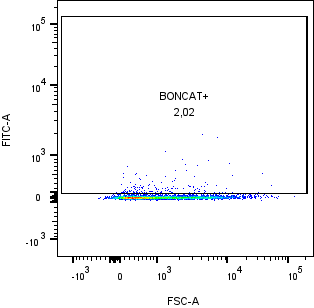


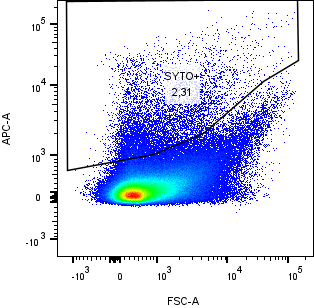

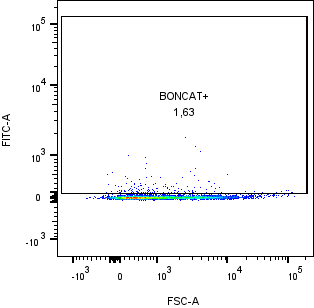


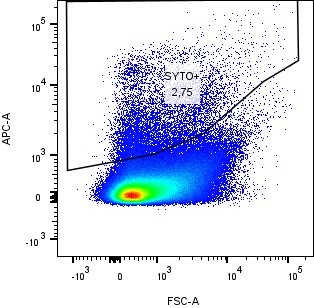

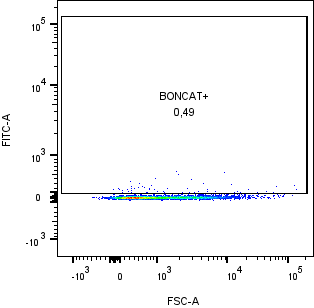


Killed control


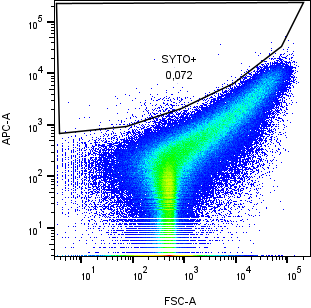

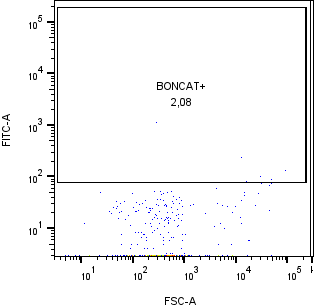


HPG-negative control (24-h water)


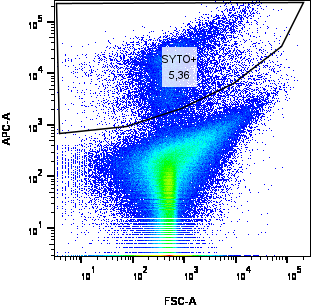

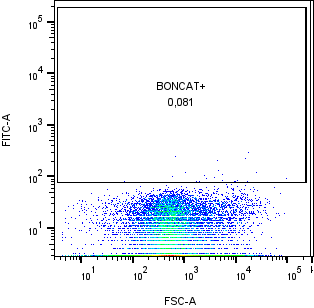


Experimental samples (12-h water)


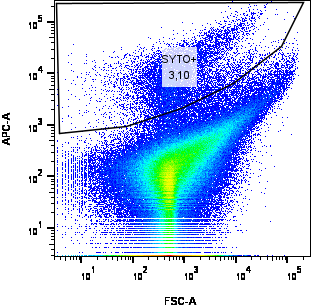

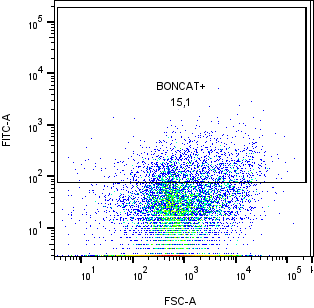


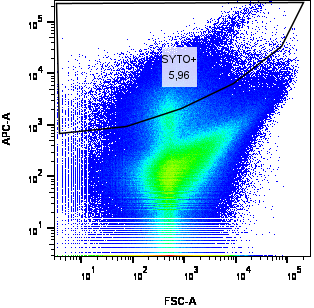

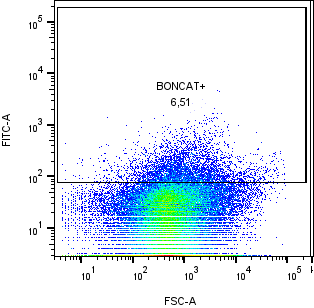


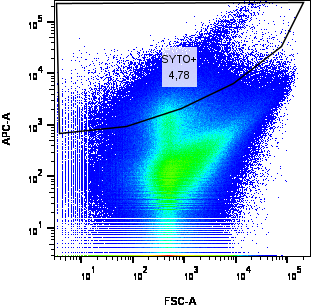

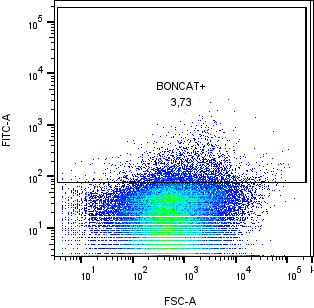


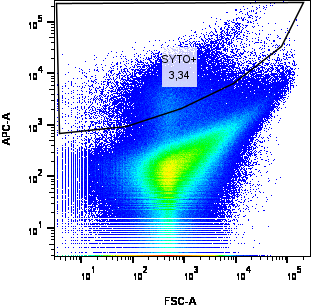

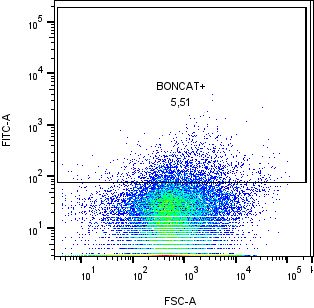


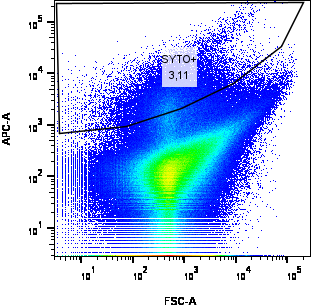

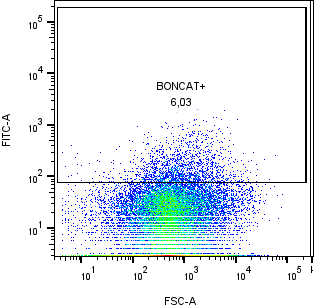


Experimental samples (24-h water)


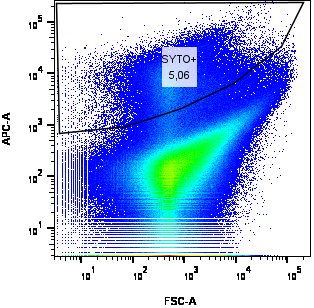

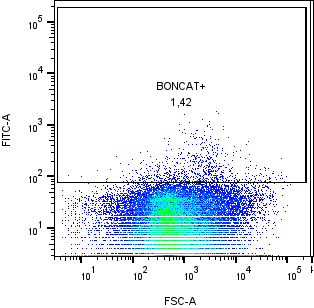


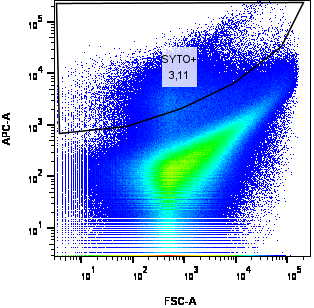

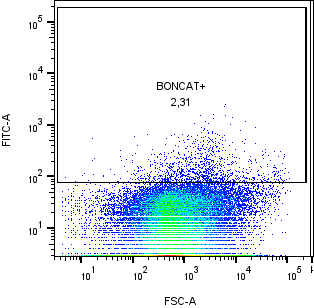


Killed control


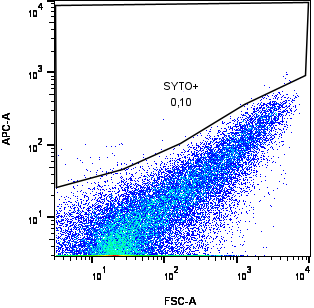

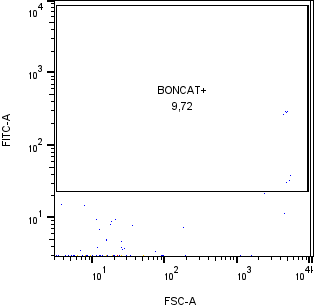


HPG-negative control (24-h water)


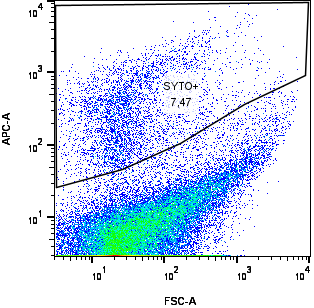

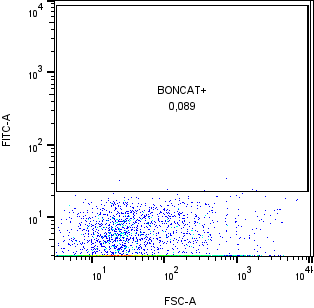


Experimental samples (24-h water)


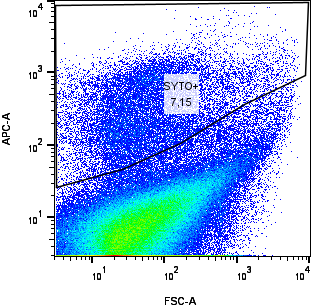

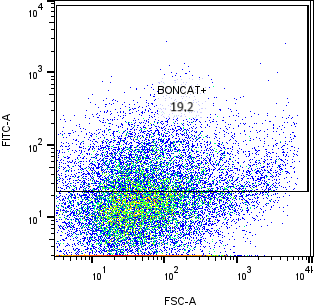


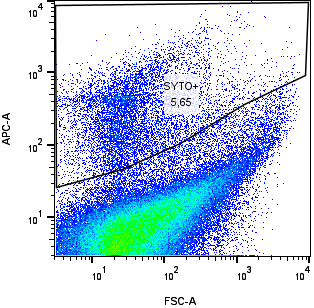

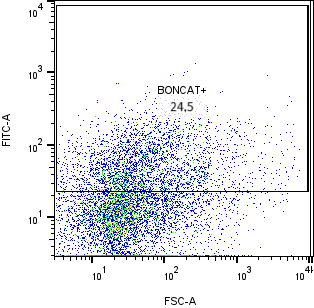


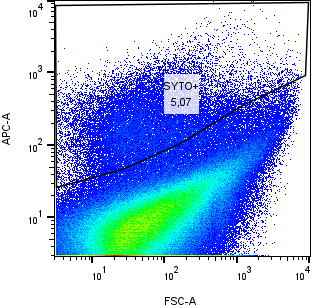

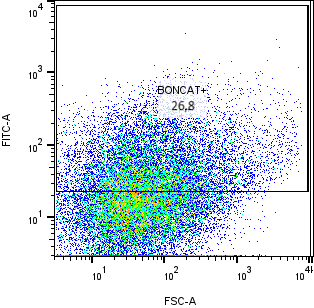


**Glucose treatment**

Killed control


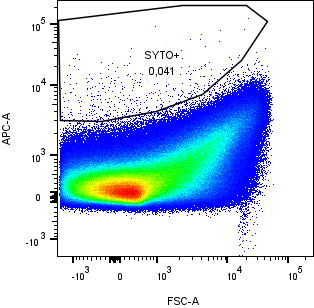

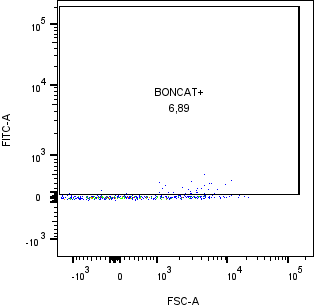


HPG-negative control (2-h glucose)


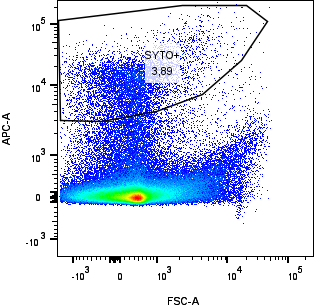

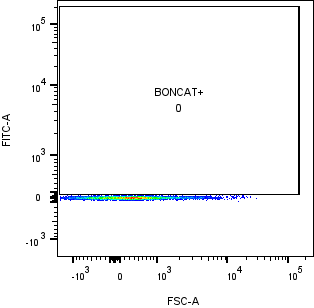


Experimental samples (2-h glucose)


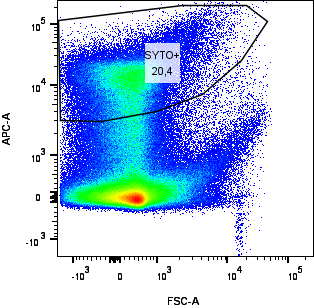

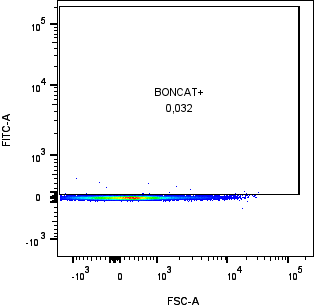


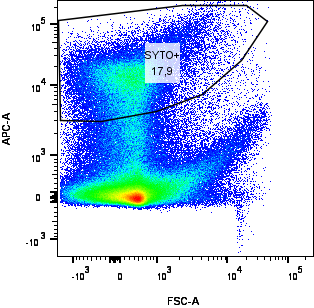

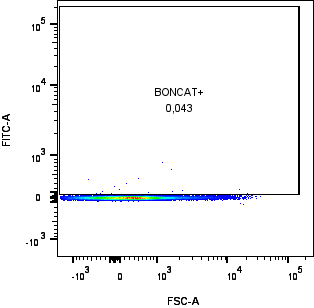

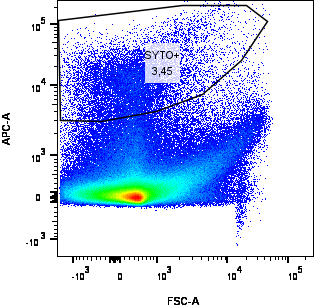

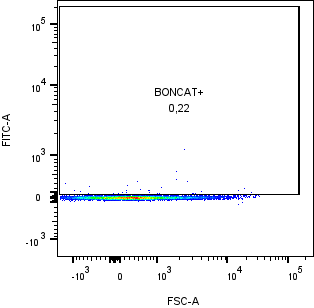


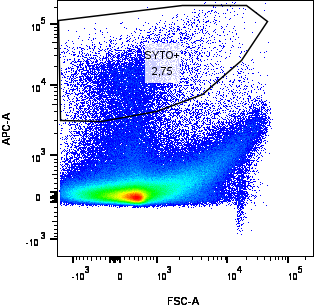

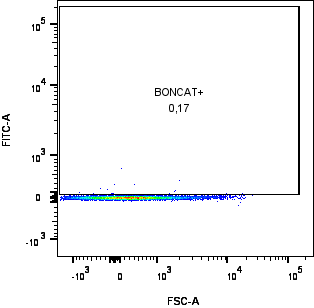


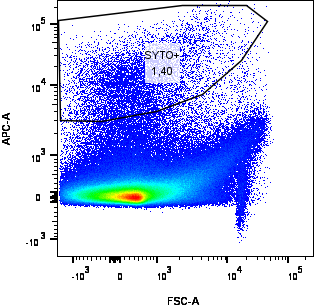

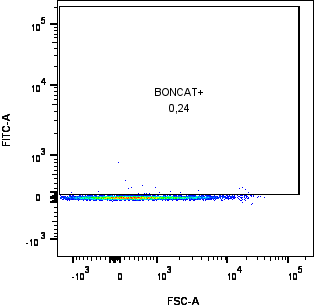


Killed control


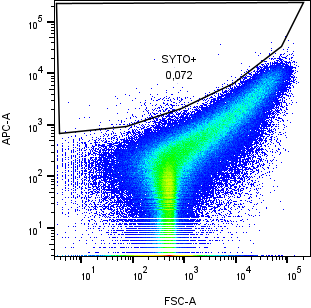

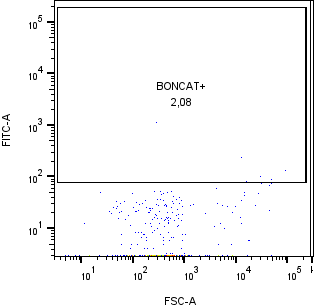


HPG- negative control (24-h water)


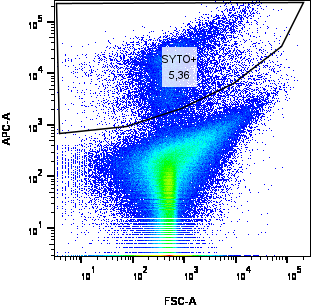

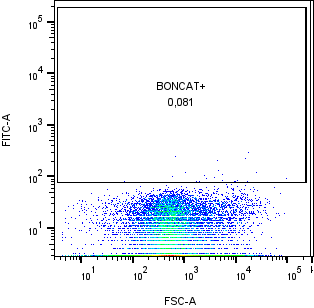


Experimental samples (6-h glucose)


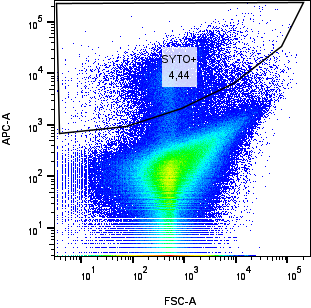

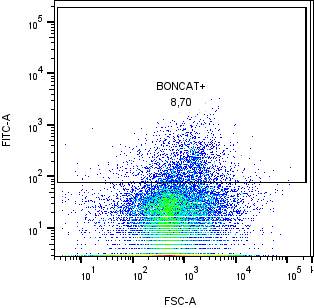


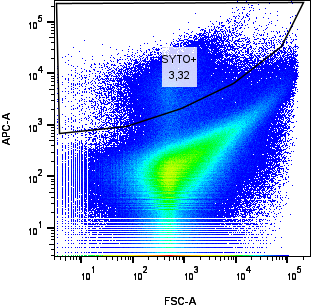

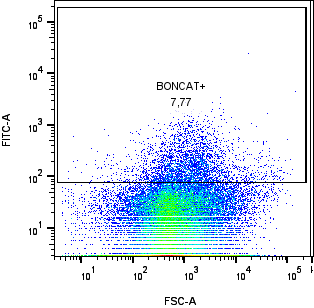


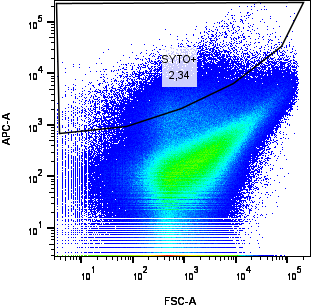

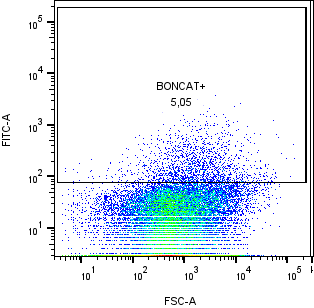


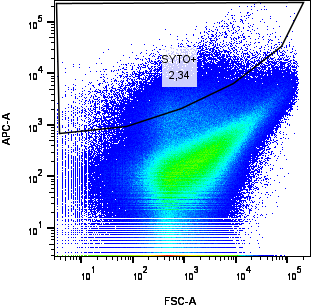

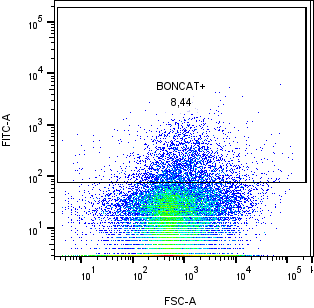


Killed control


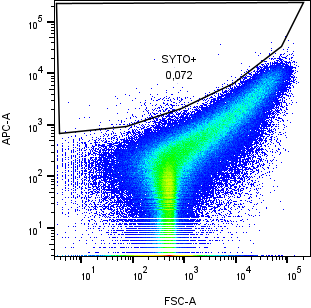

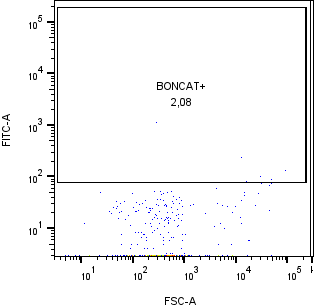


HPG- negative control (24-h water)


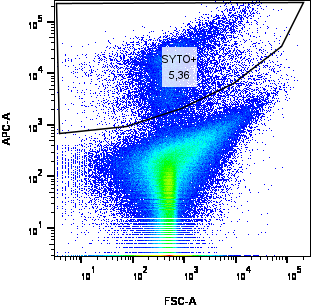

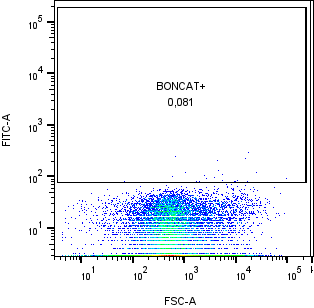


Experimental samples (12-h glucose)


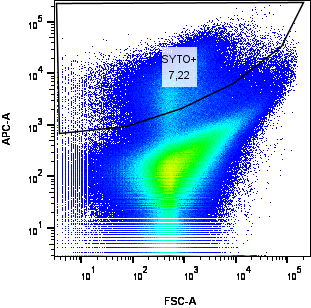

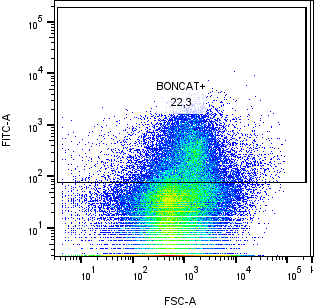


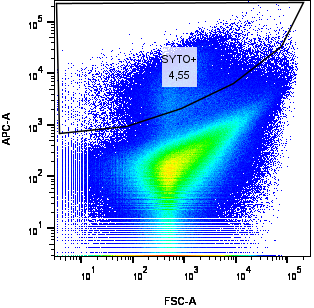

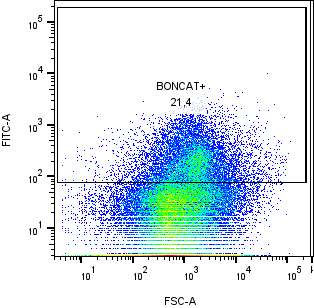


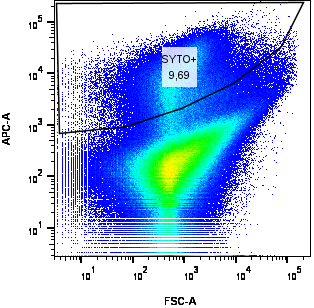

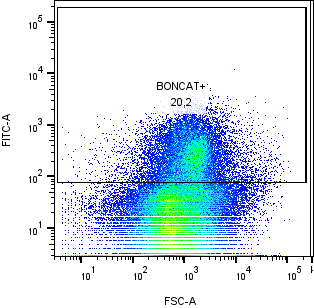


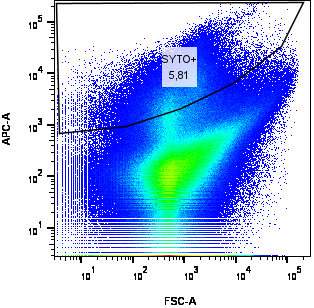

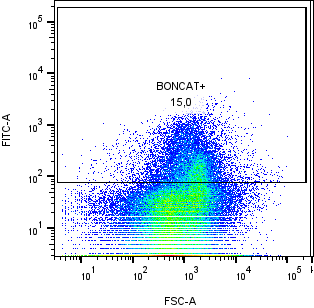


Killed control


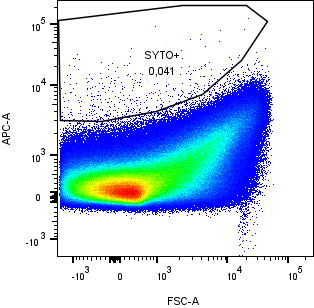

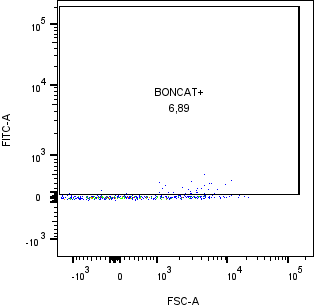


HPG-negative control (24-h glucose)


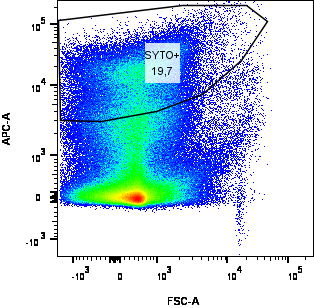

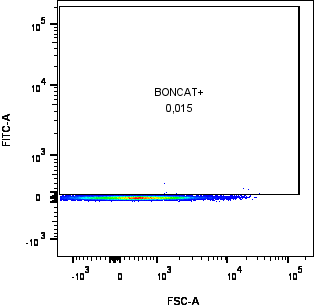


Experimental samples (24-h glucose)


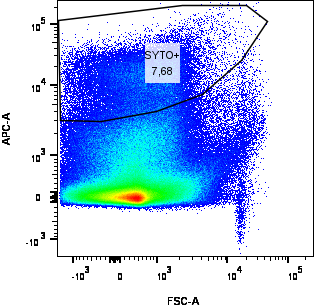

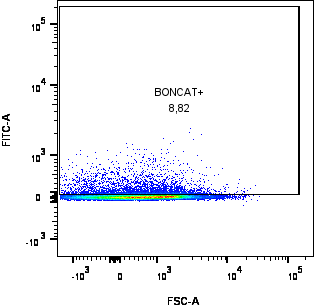


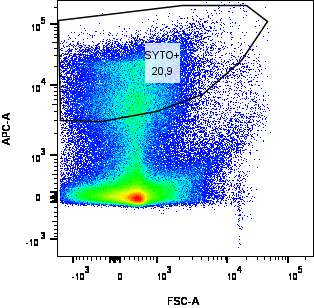

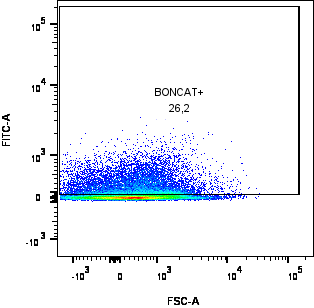


Killed control

HPG-negative control (24-h water)

Experimental sample (24-h glucose)

**Galactose treatment**

Killed Control

HPG-negative control (2-h galactose)

Experimental samples (2-h galactose)

HPG-negative control (6-h galactose)

Experimental samples (6-h galactose)

HPG-negative control (12-h galactose)

Experimental samples (12-h galactose)

HPG-negative control (24-h galactose)

Experimental samples (24-h galactose)
